# A Review on the Bioactivity of Cannabinoids on Zebrafish Models: Emphasis on Neurodevelopment

**DOI:** 10.3390/biomedicines10081820

**Published:** 2022-07-28

**Authors:** Rosario Licitra, Maria Marchese, Valentina Naef, Asahi Ogi, Marco Martinelli, Claudia Kiferle, Baldassare Fronte, Filippo Maria Santorelli

**Affiliations:** 1 Molecular Medicine and Neurobiology—ZebraLab, IRCCS Fondazione Stella Maris, 56128 Pisa, Italy; rosario.licitra@fsm.unipi.it (R.L.); valentina.naef@fsm.unipi.it (V.N.); a.ogi@hotmail.com (A.O.); 2 PlantLab, Institute of Life Sciences, Scuola Superiore Sant’Anna, 56124 Pisa, Italy; marco.martinelli@santannapisa.it (M.M.); claudia.kiferle@santannapisa.it (C.K.); 3 Department of Veterinary Science, University of Pisa, 56124 Pisa, Italy; baldassare.fronte@unipi.it

**Keywords:** zebrafish, cannabis, cannabinoids, phytocannabinoids, ∆9-tetrahydrocannabinol, THC, cannabidiol, CBD

## Abstract

For centuries, the cannabis plant has been used as a source of food, fiber, and medicine. Recently, scientific interest in cannabis has increased considerably, as its bioactive compounds have shown promising potential in the treatment of numerous musculoskeletal and neurological diseases in humans. However, the mechanisms that underlie its possible effects on neurodevelopment and nervous-system functioning remain poorly understood and need to be further investigated. Although the bulk of research on cannabis and cannabinoids is based on in vitro or rodent models, the zebrafish has now emerged as a powerful in vivo model for drug-screening studies and translational research. We here review the available literature on the use of cannabis/cannabinoids in zebrafish, and particularly in zebrafish models of neurological disorders. A critical analysis suggests that zebrafish could serve as an experimental tool for testing the bioactivity of cannabinoids, and they could thus provide important insights into the safety and efficacy of different cannabis-extract-based products. The review showed that zebrafish exhibit similar behaviors to rodents following cannabinoid exposure. The authors stress the importance of analyzing the full spectrum of naturally occurring cannabinoids, rather than just the main ones, THC and CBD, and they offer some pointers on performing behavioral analysis in zebrafish.

## 1. Introduction

For centuries, the cannabis plant (*Cannabis sativa* and *Cannabis indica*) has been used as a source of food, fiber, and medicine [1,2,3,4]. In recent decades, scientific interest in cannabis has increased considerably, as its bioactive compounds have shown promising potential in the treatment of numerous musculoskeletal and neurological diseases in humans [5,6]. Among young people, cannabis is the illicit substance that is most commonly used for recreational purposes [5,7,8], thanks to its anxiolytic effect and the associated sense of euphoria [9]. It is also widely used among pregnant women, mainly to reduce morning sickness, nausea, and vomiting [3,10]. However, because cannabinoids can readily cross the placenta and reach the fetus, they may impact the development of the embryo, which increases the risk of neurological disorders in newborns [3]. The mechanistic pathways by which cannabis and its metabolites affect neurodevelopment and nervous-system functioning remain poorly understood and need to be further investigated. To date, around 500 compounds have been identified in the cannabis plant; these include more than 150 cannabinoids, which generate more than 2000 compounds when smoked [11]. The plant also contains other bioactive compounds of medical and industrial interest, such as phenolics and flavonoids [12]. Cannabinoids and terpenes are abundant in the viscous resin that is produced by the glandular structures of the cannabis flowers, called trichromes [4]. The quantitative and qualitative characteristics of the plant are quite variable, with its composition, concentration, and yield greatly affected by the growing conditions, processing, and storage [13,14]. Biochemically, cannabinoids are highly lipophilic substances that are soluble in alcohols, fats, and other nonpolar organic solvents. They can remain associated with cell membranes long after the actual exposure to the substance [10].

In human medicine, cannabinoids are already considered to be antiemetic, antispastic, analgesic, and appetite-stimulating compounds [2,5]. Their therapeutic effects have also been examined in a series of syndromes, including multiple sclerosis [15], Dravet syndrome [16,17], epilepsy [18], fibromyalgia [19], anxiety [20], schizophrenia [21], chronic pain [22], and cancer [23,24]. Many people look to naturally derived compounds, such as cannabinoids, to treat illness and disease because they wish to avoid the strong side effects of synthetic drugs [25]. However, the literature suggests that cannabinoids may unfortunately have serious and undesirable effects, such as dependency, as well as a possible causative association with psychotic illness and cognitive impairment, including deleterious effects on memory [15]. Cannabinoids, for instance, have pronounced effects on the recognition memory and social behavior in pubertal rats, which suggests that the developing brain is sensitive to cannabinoid exposure [26]. Moreover, some studies link cannabis use to adverse birth outcomes, including low birthweights and preterm births, while other studies do not report any negative effects on children [27]. Cannabinoid use is still subject to uncertainty over aspects such as the dosing and side-effect profiles, and there is an overall lack of knowledge of their underlying mechanism of action; clinicians are therefore often reluctant to prescribe cannabis [25,28]. However, cannabis shows a lower potential to cause dependence (8.9%) than do other common substances of abuse, such as cocaine (20.9%), alcohol (22.7%), and nicotine (67.5%) [29]; moreover, it has recently been suggested that susceptibility to psychosis-like symptoms varies between cannabis consumers, as it involves a complex interplay between environmental factors and genetic predispositions [2]. Cannabinoids aside, it is also worth noting that potentially synergistic effects of phytocannabinoids and terpenoids have been reported in the treatment of pain, inflammation, depression, and anxiety [12].

Although the use of cannabis is still illegal in most countries, the cannabis world market is now approaching USD 30 billion, and the profits from illicit trafficking are certainly higher than that [30]. Currently, medications based on both synthetic cannabinoids (e.g., Nabilone^®^ and Dronabinol^®^) and cannabis extracts (e.g., Sativex^®^ and Epidiolex^®^) are approved for human use [14,16,24,31]. It is therefore very important for patients, doctors, and the entire scientific community to better understand the effects of cannabis/cannabinoid exposure on health [32].

**Figure 1:**
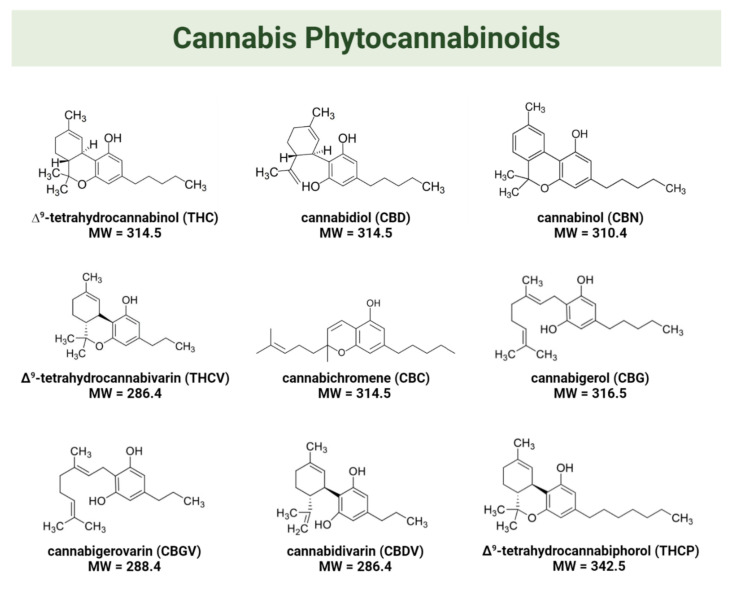
Molecular structures of phytocannabinoids found in cannabis.

The two main cannabinoids found in cannabis are ∆9-tetrahydrocannabinol (THC) and cannabidiol (CBD) [33]. THC is considered a psychoactive component, while CBD lacks psychotropic activity [10]. THC and CBD are present in the flowers and leaves of the female plants at concentrations ranging between 0.1 and 25% and 0.1 and 2.89% (*w*/*w*), respectively [30]. In addition to CBD and THC, cannabis contains numerous other cannabinoids with known or potential bioactivity [34]. For instance, cannabinol (CBN), the main metabolite of THC, was considered to be an inactive cannabinoid until studies shed light on its biological activities [30,35]. The typical concentration of CBN in cannabis inflorescences ranges between 0.1 and 1.6% (*w*/*w* of dry weight). It forms primarily through the degradation of THC that occurs as the plant ages and as an effect of storage conditions [36]. Other minor cannabinoids present in cannabis are Δ9-tetrahydrocannabivarin (THCV), cannabichromene (CBC), cannabigerol (CBG), cannabigerovarin (CBGV), cannabidivarin (CBDV), and 11-hydroxy-Δ9-tetrahydrocannabivarin (THCV−OH) [14,37]. A novel Δ9-tetrahydrocannabiphorol (THCP) was isolated and reported to have higher in vivo “cannabimimetic” activity than normal THC [38]. Figure 1 shows the molecular structures of the aforementioned cannabis phytocannabinoids.

THC and its derivatives are studied mainly for their psychotropic properties and other pharmacological activities, including their possible anticonvulsant, antidepressant, hypotensive, bronchodilator, and analgesic actions, as well as their ability to lower intraocular pressure [15]. However, there is also evidence that THC may increase the resilience to certain stressors, as it has been observed that low doses of THC protect against a wide range of neuronal insults, including 3,4 methylene-dioxymethamphetamine (MDMA) and carbon monoxide exposure [27]. In addition, researchers have shown an increasing interest in determining whether THC or other cannabinoids can positively affect neurological health and neurodegenerative disease development in advanced age. This hypothesis is supported by a study that reports that cannabinoids protect against neurodegenerative diseases in many animal models when they are administered in adulthood or advanced age [39]. The anti-inflammatory properties of THC may help to protect the brain against neurodegenerative diseases [40]. Indeed, while high doses of THC can cause memory deficits [41], low doses of THC have been shown to slow or halt Alzheimer’s disease (AD) progression by reducing the amyloid beta, which is the main component of the amyloid plaques found in the brains of people affected by AD [42,43], and to restore cognitive function in old mice [44]. Taken together, these findings reinforce the suggestion that the THC doses and patient age determine the beneficial versus detrimental effects of THC on neuronal health [27]. Moreover, the dose seems to influence the resultant behavioral phenotype, as low doses may induce anxiolytic effects, whereas high THC doses generally cause the opposite responses [45]. Some researchers suggest that the plant produces this molecule in order to protect it from ultraviolet radiation. Indeed, ultraviolet radiation can stimulate cannabinoid biosynthesis [46], and, apparently, the higher the altitude at which cannabis grows, the more THC it produces. Current evidence indicates that even visible LED light can enhance the THC, CBG, and terpene accumulation in the flowers, but not the accumulation of CBD [47].

CBD has been used to reduce bouts of nausea and vomiting, and as an anxiolytic, antipsychotic, antirheumatic, appetite stimulant, and analgesic, as well as a natural remedy for multiple sclerosis and epilepsy [10,25,48]. Moreover, CBD possesses antioxidative and antiapoptotic properties, and it exerts anti-inflammatory effects [7]. It has well-recognized behavioral effects of clinical interest, especially with regard to its anxiolytic properties, and an inverted U-shaped dose–response curve has been reported in several animal models featuring anxiety [48]. In general, CBD does not appear to affect memory formation and may protect against memory impairment [49], but research is still ongoing. Surprisingly, it has been reported that CBD degrades into THC in gastric fluid [50]. As observed with THC, the therapeutic potential of CBD is related to its interaction with the central nervous system (CNS) via several pathways, including the endocannabinoid-system (ECS) pathway, serotonin receptors, and intracellular mechanisms [25,51]. Finally, the CBD:THC ratio also seems to play an important role in determining the symptomatic effects of cannabis [6].

### 1.1. The Endocannabinoid System

In 1988, Devane and colleagues [52] discovered a specific brain receptor for cannabinoids. Subsequently, it was understood that the cells equipped with these receptors constitute part of a network of neurons, analogous to that involving dopamine, serotonin, and endorphins, and capable of triggering cognitive, behavioral, or physiological changes. Cannabinoid receptors were found to be particularly expressed in brain areas involved in the control of learning and memory (cortex and hippocampus), motor behavior (basal ganglia, cerebellum), emotions (amygdala), and autonomic and endocrine functions (hypothalamus, pons, and medulla), and they may therefore be involved in the control of numerous neurobiological processes [53,54]. Four years after the discovery of cannabinoid receptors, the same research group [55] isolated an endocannabinoid produced by the human brain. It was named “anandamide” (AEA), after the Hindu term “Ananda”, meaning “happiness or bliss”. Specifically, there is evidence of a role for AEA in social facilitation, which is closely related to the action of oxytocin [56]. Indeed, the oxytocinergic system is known to regulate social and maternal behavior in mammals [57], and AEA, mediating the action of oxytocin, has been considered crucial for social behavior, and even a possible therapeutic compound for autism-related social impairment [58]. Although the scientific literature on the relationship between zebrafish social behavior and isotocin (teleost homolog of mammalian oxytocin) is quite limited, the effects of bioactive fatty acid amide derivatives on zebrafish bone metabolism [59], growth, and lipid metabolism [60] have been described.

In vertebrates, the ECS involves the cannabinoid receptors 1 and 2 (CB^1^ and CB^2^, re-spectively), endogenous ligands (such as anandamide and 2-arachidonoylglycerol), and the mechanism responsible for receptor and ligand synthesis and degradation [10]. CB^1^ has been found to be the most abundant G-protein-coupled receptor within the CNS [5]. CB^1^ and CB^2^ are activated both by endogenous ligands and exogenous phytocannabinoids, such as THC and CBD [10]. These receptors are typically located presynaptically, and they work as retrograde messengers to decrease the synaptic output. By activating the G_α_ subunit, cannabinoids are able to inhibit voltage-gated calcium channels and potentiate inwardly rectifying potassium channels [61]. Even though CBD interacts with both cannabinoid receptors, it shows lower affinity compared with THC [3]. Indeed, CBD seems to be 10 times less active than THC on both CB^1^ and CB^2^ [48]. CB_1_ controls the vesicular release of gamma aminobutyric acid (GABA) or glutamate by inhibiting voltage-gated Ca^2+^ channels [62]. Moreover, CB_1_ is also present in the external membrane of mitochondria [63], where it regulates memory processes via the modulation of the mitochondrial energy metabolism [64]. In addition, several findings have shown that the ECS, through CB_1_ receptor activation, is associated with the neuronal differentiation and maturation of adult progenitor stem cells into neurons or astrocytes [65], which is a role that could be relevant in the treatment of neurodegenerative diseases. Conversely, although CB_2_ expression was initially described only in the immune system, more recently, it was also detected in particular brain regions [66], and previous studies in rodents have already reported schizophrenia-related behaviors [67,68], altered cognitive function [69], modified drug-reward behaviors [70], and increased aggressiveness and anxiety [68] in CB_2_-knock-out mice. By contrast, CB_2_ overexpression was associated with reduced anxiety-like behaviors and higher resistance to depression in a murine model [71,72]. In this context, it has been suggested that CB_2_ can regulate the synaptic transmission in hippocampal pyramidal cells and modulate both the gamma oscillation and activity of the sodium–bicarbonate co-transporter, which leads to a hyperpolarization of the neurons [73]. The ECS has also been shown to modulate the expression of neurotransmitters in the basal ganglia that is involved in coordinated movement [74], and it has the ability to control neuronal migration and differentiation by regulating growth-factor activities [10,75]. Through the activation of their receptors, cannabinoids can regulate synaptic neurotransmission, playing a key role in AD, anxiety, epilepsy, multiple sclerosis, Huntington’s, and pain perception [39,53,66,76]. While most actions of cannabinoids are mediated through the activation of CB_1_ and CB_2_, cannabinoids can produce effects completely or partially independent of the aforementioned receptors, acting instead through other G-protein-coupled receptors, such as GPR18 and GPR55, serotonin receptors (5HT1Rs), and vanilloid transient receptor potential cation channel receptors [10,24], as well as receptors of the dopaminergic, glutamatergic, cholinergic, and opioidergic systems [2,45].

### 1.2. Zebrafish as a Model System to Test the Bioactivity of Cannabinoids

Although the bulk of the literature published to date on cannabis and cannabinoids consists of experiments performed using in vitro or rodent models, the zebrafish (*Danio rerio*) has recently gained attention as a powerful in vivo model, combining the experimental efficiency of cell cultures and organoids with the opportunity to study whole living vertebrate organisms [77]. Over the past three decades, the use of zebrafish has helped to further the knowledge and understanding of the neurobiological basis of vertebrate behavior and the pathogeneses of human neurological diseases [2,25,78,79,80]. Zebrafish show high genetic homology to mammals; the sequencing of the zebrafish genome revealed that 70% of human genes have at least one zebrafish ortholog, and that 84% of genes known to be associated with human disease have a zebrafish counterpart [17]. Many zebrafish genes are duplicated, making the investigation of their functions particularly challenging [81]. One advantage of zebrafish as a model species is that their embryos develop externally, which facilitates the study of embryo development [15]. Zebrafish development progresses quite quickly, with most organs developed within the first hours postfertilization (hpf); muscle activity starts from 17 hpf [81]. Pharmacological screening is among the most common applications of zebrafish [45,77]. At all stages of development, zebrafish can absorb through the skin’s small molecules from the surrounding water, and this makes them ideal for performing studies on drug bioavailability and metabolites in a multiorgan system [24]. Moreover, numerous genetic tools, in vivo imaging techniques, and electrophysiological and neurobehavioral assays can be used to study the consequences of drug administration in zebrafish [82,83,84,85]. The ECS is highly conserved between zebrafish and mammals—this is not a characteristic of common high-throughput invertebrate model organisms—and ontogenetic analysis has revealed that ECS gene expression begins early during zebrafish development [79]. Recently, the zebrafish ECS has been well characterized: it comprises the same receptors, ligands, and enzymes as its mammalian equivalent [86,87]. Zebrafish larvae begin to express *CB_1_* mRNA at the three-somite stage; expression is widespread in the CNS (preoptic area, telencephalon, hypothalamus, tegmentum, and anterior hindbrain) at 48 hpf, with the highest expression occurring in the telencephalon at 96 hpf [31,86,88]. In addition, CB_1_ protein has been observed in larval zebrafish brain homogenates from 48 hpf through 15 days postfertilization (dpf) [88]. A high level of sequence conservation of CB_1_ has been shown between zebrafish and mammals. Indeed, the receptor shows 65–69% similarity at the nucleotide level, and 66–75% at the amino acid level [89]. One study showed that the morpholino knockdown of the *cnr1* gene (encoding CB_1_) led to aberrant patterns of axonal growth and the fasciculation of reticulospinal neurons [90]. These data support the idea that CB_1_ is needed for brain and locomotor behavior development, even in fish larvae [88]. Less is known regarding the *CB_2_* expression patterns throughout zebrafish development, but a comparison of the zebrafish CB_2_ revealed a 39% amino acid similarity with its human counterpart [17]. Elsewhere, after the generation of a CB_2_-knock-out zebrafish, the resulting homozygote (*cnr2 ^upr1/upr1^*) larvae were shown to be characterized by lower swimming performances and increased anxiety-like behaviors [66]. These findings suggest that zebrafish could be a suitable model for investigating individual ECS gene functions, and for identifying novel genetic modifiers of cannabinoid signaling. Recently, zebrafish were used to test the effects of cannabinoids, administered alone, in combination, and as part of a complex, and were found to offer certain distinct advantages over mammalian models for drug studies [91,92]. The exposure of zebrafish to cannabinoids has been shown to alter a range of behaviors, physiological processes, and gene-expression pathways that are closely related to the ECS [75]. A broad range of behaviors can be analyzed in zebrafish larvae, including multiple swimming parameters, optokinetic and optomotor responses, prey tracking, phototaxis and thigmotaxis, and even learning and memory [93,94]. Due to the rapid development of larvae, these behaviors can be studied within the first week after fertilization. To evaluate the behavioral effects of cannabinoids on zebrafish larvae, most researchers have used the visual-motor-response (VMR) test, which is a validated behavioral assay that measures larval activity first in a light environment, and then in darkness, to study a single transition or dark–light cycles [15,25,31,32]. Typically, zebrafish larvae make frequent low-amplitude movements when exposed to a stable light condition, but an abrupt transition from light to dark causes an immediate increase in their motor activity for 10–15 min, after which it slowly declines to baseline levels [78,86,95,96]. The VMR test has been used to evaluate the sensory-motor function of zebrafish mutants/transgenic lines, and to assess the neurobehavioral responses to nutraceuticals and drugs [78,91]. This behavioral assay thus makes it possible to assess the effects of each compound both on baseline activity and after a standardized stimulus.

To assess the zebrafish anxiety state, and the related efficacy of anxiolytics, two behavioral assays are commonly used: the thigmotaxis paradigm, which is based on an analysis of the preference to swim in close proximity to the tank walls [32], or the light–dark preference test, which is based on the known marked preference of zebrafish larvae for the dark compartment [97]. In the latter test, an increase in activity and time spent in the white/light compartment is considered to reflect anxiolytic behavior, whereas increased activity in the dark compartment indicates anxiety-promoting behavior. Adult zebrafish, due to their size and low housing costs, also provide a cost-effective model for molecular-screening purposes. The most popular, sensitive, and reliable behavioral test in adult zebrafish is the novel tank paradigm, in which the fish locomotor activity and anxiety can be monitored at the same time [98]. Behavioral phenotypes in adult zebrafish are already well characterized [94] and include social, aggressive, affective, and cognitive behaviors [99,100,101], which are all highly sensitive to a wide range of CNS drugs [102].

We here review the available literature on the use of cannabis/cannabinoids in zebrafish models in order to establish, through a critical analysis of the articles, whether zebrafish might serve as a powerful experimental tool for testing the bioactivity of cannabinoids, and thus for gaining important insights into the safety and efficacy of different cannabis-extract-based products.

## 2. Materials and Methods

### Data Sources and Searches

**Figure 2:**
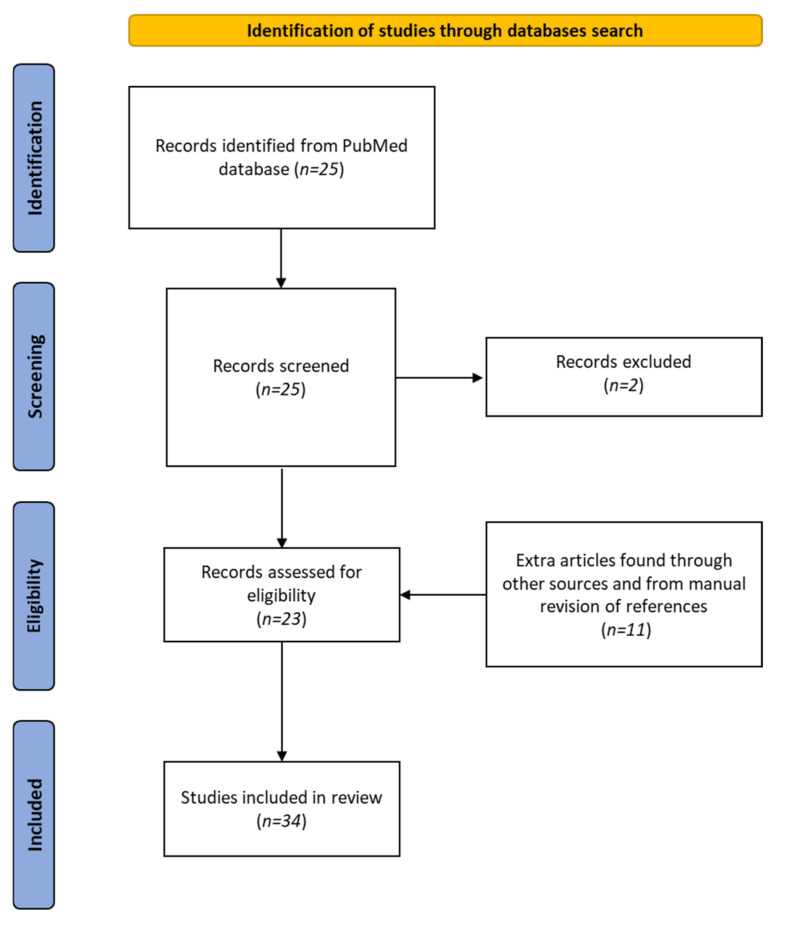
PRISMA 2020 flow diagram of the literature-search process.

We followed the Preferred Reporting Items for Systematic Reviews and Meta-Analyses (PRISMA) guidelines. The study was registered in PROSPERO; registration number was 344190. The search was conducted by a medical librarian in MEDLINE (via PubMed up to 20 December 2021) using the keyworks “cannabis” (all fields) AND “cannabinoids” (all fields) AND “zebrafish” (all fields). The search yielded 25 matches, but 2 articles were excluded: one because the authors did not discuss the effects of cannabis on zebrafish, and the other because it did not concern zebrafish. The reference lists of these publications were examined, and a further 11 papers were identified. Overall, 34 articles were included in this review. Figure 2 shows a PRISMA flow diagram summarizing the methodology, which was created following the recent indications of Page et al. [103]. 

Table 1 gives details of the experimental protocols of all the studies included in the review. It must be emphasized that the single cannabinoids tested were purified standard chemicals, which were used in all the studies, except for one, where THC was purified by using centrifugal partition chromatography [15]. Whereas in the two studies in which the whole-plant cannabis extract was employed, the analytical determination of the main cannabinoids was performed by gas or liquid chromatography coupled with high-resolution mass spectrometry.

## 3. Discussion

### 3.1. Effects of Phytocannabinoids in Wild-Type Zebrafish

The use of zebrafish to test the toxicity of phytocannabinoids dates back to a 1975 study in which THC was dissolved in aquarium water (acute exposure), and its median lethal dose (LD_50_) calculated in zebrafish embryos was found to range between 2 and 5 mg/L [104]. Interest in studying cannabis/cannabinoids in the zebrafish model, however, has grown only in the past 10–15 years. The harmful effects of cannabinoid administration during zebrafish embryonic development have been well studied: embryos treated with THC and/or CBD exhibited shorter body lengths and mild deformities, reduced survival and basal heart rates, decreased synaptic activity and red-muscle-fiber thickness, alterations in the branching patterns of secondary motor neurons and Mauthner cells, changes in the expressions of postsynaptic nicotinic acetylcholine receptors in skeletal muscle, and reduced hatching rates [10,15,75]. In these studies, THC and CBD were used at concentrations believed to mimic the physiological range of cannabis use in humans (0.3–10 mg/L and 3–4 mg/L, respectively). In this regard, blood-plasma concentrations of THC and CBD caused by the consumption of a single cannabis cigarette have been found to reach peaks as high as 0.162 and 0.056 mg/L, respectively [105,106]. Table 1 summarizes studies on this topic.

Considering the deleterious effects of THC and CBD on developing embryos, the impact of these compounds on neural activity has recently been investigated through a novel in vivo assay based on a calcium-modulated photoactivatable ratiometric integrator (CaMPARI) system, which is able to provide a practical read-out of the neural activity in freely swimming larvae [3].

**Figure 3:**
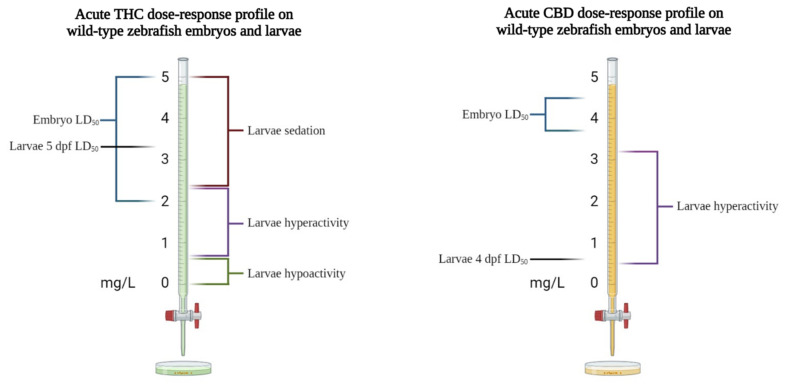
Toxicological and behavioral effects of acute THC and CBD administration on wild-type zebrafish embryos and larvae.

In acute regimens, both THC and CBD, if administered at high concentrations (6 and 3 mg/L, respectively), dramatically reduced the neural activity and locomotor activity of larvae at 4–5 dpf. Interestingly, the neuro-locomotor decrease was more pronounced when CBD and THC were combined. When treating embryos and 4 dpf larvae with low concentrations of CBD (up to 0.3–0.6 mg/L), no significant differences in the morphological parameters were observed, although the CBD significantly delayed the hatching of the embryos at the highest concentration used [32,51]. In most behavioral studies on the effects of cannabinoids in zebrafish, larvae were used at 5 dpf because, at this stage, they have fully developed digestive systems and inflated swim bladders, show mature swimming, and actively search for food [81,107]. In wild-type larvae at 5 dpf, the LD_50_ for THC, measured after chronic exposure (96 h beginning at age 24 hpf), was 3.37 mg/L [15]. In a study using zebrafish larvae with different characteristics and considering different drug-exposure times, a similar THC LD_50_ (3.65 mg/L) was found in fluorescent zebrafish of the Tg(fli1: EGFP) transgenic line at 4 dpf [86]. In acute regimens, the exposure of wild-type larvae to THC prompted a biphasic behavioral response consisting of increasing hyperactivity at concentrations ranging from 0.6 to 1.2 mg/L (2–4 μM), followed by the suppression of activity as the dose increased to 3.4 mg/L (10.8 μM) [15]. In line with these results, younger larvae (4 dpf) exposed to 0.3 mg/L THC exhibited a significantly increased duration of movement, while doses in the 0.6–1.25 mg/L range reduced the locomotor activity [32,86]. Evidence for the sedative effect of high doses of THC is also provided by Thornton et al. [14] and Amin et al. [75], who showed that THC at concentrations of 4–6 mg/L reduced swimming performances. These findings are consistent with results reported in rodents (i.e., dose-dependent hyperactivity followed by suppression at higher concentrations), as well as with the well-reported “stoning” action of THC in humans [45,108]. In chronic regimes, THC showed habituation, which is the development of tolerance to many of the acute effects in chronic exposition. Nevertheless, THC at 1.2 mg/L increased the distance traveled by fish [15]. This phenomenon has been associated with the downregulation of cannabinoid receptors after long-term exposure to cannabinoids [109]. In addition, the observation of reduced larval basal activity in response to exposure to THC at doses of up to 0.625 mg/L (2 µM) [31] suggests that THC produces a calming effect on larval locomotor activity up to this concentration, as opposed to hyperactivity at concentrations ranging from 0.6 to 2.4 mg/L, and sedation at concentrations higher than 2.4 mg/L (see Figure 3). In this context, psychoactive drugs, such as THC or its analog WIN55,212-2, by activating cannabinoid receptors, can induce hypothermia and hypoactivity, increase tremors and startle behaviors, and, in severe cases, induce catalepsy-like immobilization [110,111].

Zebrafish treated with WIN55,212-2 at 0.5 and 1 μg/mL showed no activity, even in darkness, whereas this was lethal if applied at 10 μg/mL [111]. Chronic early-life treatment with THC (0.6 mg/L) did not affect the locomotor abilities in 30-month-old zebrafish, which suggests that this psychoactive cannabinoid has no long-term effects on swimming behavior if used at low doses [27].

As for CBD, embryonic exposure to concentrations of up to 0.15 mg/L did not cause notable morphological abnormalities [32]. The LD_50_ values for CBD, calculated in zebrafish, are 4.4 mg/L at 2 dpf, 3.7 mg/L at 3 dpf [112], and 0.53 mg/L at 4 dpf [86]. In this latter study, larvae chronically exposed to low concentrations of CBD showed a biphasic locomotor response pattern, similar to that previously reported for THC [15]. In detail, 0.07 mg/L CBD produced a significantly increased duration of larval movement, while concentrations of 0.1–0.3 mg/L had a hypolocomotor effect. The acute administration of CBD at doses of up to 0.3 mg/L did not alter the locomotor behavior of 5 dpf zebrafish larvae, whereas higher concentrations caused larval hyperactivity [31]. In support of these findings, a study using auditory/mechanical tests to evaluate fish behavioral responses to unexpected sound and touch stimuli showed that THC and CBD concentrations of 6 mg/L and 3 mg/L, respectively, reduced their responses to sound [10]. An inhibitory effect on locomotion of low doses of CBD, ranging from 0.5 to 10 μg/mL, has been reported, but without a dose-dependent mechanism [111]. The same research evaluated larval responses to CBD after an initial exposure to WIN55,212-2. The results indicated that CBD could attenuate the WIN55,212-2-induced abnormal immobilization. Differences between the control and CBD-treated groups were no longer detected after 24 h of recovery in clean water, and this recovery trend was observed even after exposure to toxic levels of WIN55,212-2. Another study tested the analgesic properties of THC and CBD in a zebrafish larval model of nociception [25]. In detail, larvae, while recovering from acute exposure to low levels (0.1–0.5%) of acetic acid (nociception stimulus), were exposed to low levels of THC or CDB (0.15 mg/L). The THC-exposed larvae showed reduced activity compared with that of both the acetic acid-treated and control groups, which is in line with the proposed calming effect of THC at doses of up to 0.6 mg/L (Figure 3). Notably, however, CBD appeared to increase the larval locomotor activity after acetic acid exposure, and it also had a nominal effect on the control-group locomotion, seemingly confirming its nociceptive properties. In other research analyzing both the immediate and long-term effects of THC (up to 0.6 mg/L) and CBD (up to 0.15 mg/L) on larval locomotor behavior, it was observed that THC exposure reduced the swimming behavior in the treated larvae (F0), as previously reported, whereas the locomotor parameters in their offspring (F1) were increased in comparison with the controls. Instead, CBD had no effect on F0 larvae, and it decreased activity in unexposed F1 larvae [32]. Furthermore, in 3 dpf larvae, 1.25 mg/L of CBD extract accelerated the caudal-fin regeneration and reduced apoptosis after amputation [112].

Several different cannabinoids have been tested on 5 dpf wild-type zebrafish larvae. In particular, exposure to CBN and CBDV at concentrations higher than 0.75 mg/L led to malformations and bradycardia, and the calculated LD_50_ for CBN was 1.12 mg/L [14,30]. A behavioral analysis suggested that the locomotion of the treated larvae remained unaltered up to 0.043 mg/L of CBN [14], but was significantly reduced at higher concentrations, in both dark and light conditions, which also affected their anxiety status. Conversely, CBDV administration had no significant effect on zebrafish [30]. In another study, a novel dihydrophenanthrene derivative, isolated from commercial cannabis, exhibited behavioral dose effects similar to those previously described with CBD [12]. Evaluating the toxicity and antitumor effects of abnormal CBD and its analog O-1602 (which have no or only little affinity for CB_1_ and CB_2_), Tomko et al. [24] found that both atypical cannabinoids significantly reduced tumor growth, but concentrations greater than 0.8 mg/L caused higher levels of toxicity to the larvae. Finally, data from another study indicated that THCV and THCV−OH have significant effects on the skeletal ossification of larvae at 8 dpf [37].

Recently, two similar behavioral studies, conducted independently in Canada and Italy, evaluated the effects induced by full-spectrum cannabis extracts, as opposed to purified major cannabinoids, on the zebrafish model. Research data on these extracts are scarce, and because cannabis consumers use the entire inflorescences, more scientific evidence is needed to clarify the bioactivity of all the cannabinoids, including their simultaneous interactions. In the study by Nixon et al. [92], acute exposure to the extracts produced similar complex concentration-dependent activity patterns to those observed by the group when using pure THC and CBD in a previous study [31]. However, distinct concentration-dependent differences were found both between the extracts (characterized by different ratios of THC:CBD) and versus the purified THC and CBD, which suggests that these differences might be related to the activity of other minor cannabinoids (specifically CBC, CBG, and CBDA). In the study by Licitra et al. [91], an excitatory effect on the locomotor activity was observed in larvae exposed to cannabis extract derived from CBD-rich-strain plants (containing about 0.5 and 7 μg/L of THC and CBD, respectively), without leading to toxicity effects. These studies underlined that the precise bioactivity of the single compounds in cannabis extracts and their interaction with the ECS pathway are highly complex issues that require further work.

Research on acute exposure to the cannabis receptor agonists WIN55,212-2 and CP55,940 indicated that both compounds reduce the locomotor activity in a dose-dependent fashion, in both light and dark phases, while the specific CB_2_ agonists HU-910 and JWH-133 had no effect on locomotion, in any circadian phase [87]. Using cnr1^−/−^ larvae, the authors found no inhibitory effect of WIN55,212-2 or CP55,940 on the average swimming velocity. The CB_1_ antagonist AM251 did not affect locomotor activity, but blocked the effect of WIN55,212-2, which indicates that these endocannabinoids are not active in regulating the locomotor activity in zebrafish larvae at 5 dpf.

Another gene-expression analysis, performed on 4 dpf fluorescent larval zebrafish exposed at 96 hpf to THC or CBD, focused on the differential expressions of 10 key morphogenic or neurogenic genes [86]. The authors found the c-fos expression to be differentially upregulated in a concentration-dependent manner following both THC (1.25 and 2.5 mg/L) and CBD (0.07 and 0.1 mg/L) exposure, and it was correlated with increased neural activity and hyperlocomotor behavior in the zebrafish. In addition, the same concentrations of THC resulted in deleted in azoospermia-like (dazl)-gene upregulation, while the expressions of vasa, sox2, sox3, sox9a, bdnf, reln, krit1, and the CB_1_-expressing gene cnr1 were similar to the control values. Along the same lines, during the key developmental stages (14, 24, 48, 72, and 96 hpf), THC and CBD caused the differential expressions of c-fos, bdnf, and dazl [32]. Contrary to the findings on cnr1 gene expression reported by Carty et al. [86], treatment with a full-spectrum cannabis extract (THC-poor strain) induced the overexpression of both cnr1 and cnr2 cannabinoid receptors in 5 dpf zebrafish larvae [91]. Additionally, CBD was found to reduce the gene- and protein-expression levels of cxcl8, tnf-α, and il-1β, and of IL-1β, caspase 3, and PARP [112]. Pandelides and colleagues observed that treatment with cannabinoids can alter the expression of proinflammatory cytokines in aged fish, which suggests a possible reduction in inflammation over the course of the lifespan [27]. In particular, exposure to low levels of THC during zebrafish development led to a significant reduction in tnf-α and il-1β at an advanced age, but this was not observed at higher doses, which indicates a biphasic or hormetic effect. Furthermore, the differential effect on the pparγ expression of exposure to cannabinoids in adult male vs. female zebrafish suggests that cannabinoid exposure could have long-term effects on reproduction, growth, and survival during early development [27].

In addition to larval locomotor activity, Achenbach et al. [31] assessed the uptake kinetics of THC and CBD, and their possible metabolism by larvae, suggesting that both cannabinoids are bioaccumulated in the living organism, but at concentrations that are substantially lower than their levels in test media. Studies involving liquid chromatography–tandem mass spectrometry analysis have shown that, when a test compound is dissolved in the embryo water, only 0.1–10% of it typically crosses the chorion and actually reaches the embryo [3,10], which limits the effectiveness of the treatment. In support of this, Carty et al. [87] found that, despite the best laboratory efforts, the actual THC concentrations in water corresponded to between 64% and 88% of the expected values at time 0, and the THC detection rate fell to between 16% and 32% at 96 hpf. Similarly, the actual CBD concentrations were only 33–40% of the nominal at time 0, and decreased to either not detected or 3% of baseline after 96 h. Indeed, in pharmacological and toxicological research with aquatic species, where the test compounds are usually diluted in the incubation water, it is essential to consider the relationship between the drug concentration in the medium and its adsorption and degradation rates.

Behavioral data from adult wild-type zebrafish indicate that a sedative effect was evoked following acute exposure to high doses of THC (30 and 50 mg/L) [45]. Moreover, reduced top swimming behavior was observed during the THC exposure, which indicated an anxiogenic effect. In another study, low doses of THC (up to 0.6 mg/L) did not cause significant behavioral effects in treated adults, but a significant reduction in thigmotaxis was seen in F1-generation fishes [32]. Other authors, however, have found these THC doses (0.3–0.6 mg/L) to induce repetitive swimming patterns in adult zebrafish [2]. In the same study, N-methyl-D-aspartate (NMDA), GABA antagonist pentylenetetrazol (PTZ), selective CB_2_ inverse agonist AM630, and sulpiride (an antipsychotic) attenuated a THC-induced behavioral stereotypy, while the selective CB_1_ inverse agonist AM251 did not. These results support a possible role for CB_2_ as a mediator of abnormal behavioral patterns induced by THC [2]. In terms of cognitive abilities, it has been reported that the acute administration of tiny doses of THC (0.03 mg/L) did not lead to any observable effect on color-discrimination learning, but heavily impaired the fish spatial-memory retrieval [113]. Conversely, in studies of possible CBD effects, acute exposure to 40 mg/L reduced the swimming speed and distance [7], while no changes in these parameters were reported when using concentrations ranging from 0.1 to 10 mg/L [48]. These latter lower doses showed an anxiolytic effect on zebrafish in the novel tank test, which is in line with the findings in acute regimens in mammalian models [114]. However, CBD at 5 mg/L caused memory impairment in an avoidance task, while the same dose did not affect aggressive behavior and social interaction [48].

Studies exploring the reproductive effects of cannabinoids suggest that developmental exposure to THC can cause persistent sex-specific alterations to the reproductive system, particularly in male fish [32], and even across generations [27]. Similarly, THC treatment significantly reduced the ATP levels in mammal spermatozoa [115], and altered ECS signaling is linked to infertility in human semen [116]. Thus, the reproductive effects could be a result of altered metabolism [27]. In rodents, greater tolerance to THC in female rats than in male rats has been observed; this is probably due to the presence of hormones that are able to modulate the THC effects [117]. However, both in rats and zebrafish, maternal exposure to THC has been linked to altered locomotor and exploratory behavior in the offspring [32,118]. Unexpectedly, treating embryos with a low dose of THC (0.024 mg/L) increased the survival in aged males (30 months old), while, in aged females, the same dose improved egg production and reduced body mass [27].

### 3.2. Effects of Phytocannabinoids in Zebrafish Models of Neurological Disorders

The observed neuromodulatory effects of cannabinoids on the CNS have led neuropharmacological researchers to increasingly focus on the clinical potential of these molecules for use in the treatment of neurological disorders. Several zebrafish lines characterized by neuro-hyperactivity, seizures, bipolar disorder, and anxiety/stress and addiction behaviors have already been developed [17,31,119]. Epilepsy is a common neurological disorder that affects over 70 million people worldwide [120]. Approximately one-third of patients show multidrug resistance [121]. Therefore, research efforts are aimed at developing new drug treatments. Seizure treatment is one of the oldest reported uses of cannabis, and recently, the use of pure cannabinoids has been suggested as a means to treat severe forms of refractory childhood epilepsy (i.e., Dravet syndrome) [122,123]. Several zebrafish models of epilepsy, and more generally of psychiatric and muscular disorders linked to neuro-hyperactivity, have already been created and offer several specific key advantages, as explained below [14,17,119]. A number of small molecules targeting different receptors or ion channels can be used to induce seizures or neural hyperactivity in zebrafish larvae. The best characterized chemically induced model is PTZ exposure. Zebrafish larvae exposed to PTZ show a concentration-dependent abnormal pattern of behavior: increased locomotion followed by fast darting activity, and finally, clonic convulsions accompanied by a loss of posture [119]. In addition, PTZ administration leads to electrophysiological changes in the zebrafish optic tectum [124]. For instance, homozygous scn1Lab^−/−^ mutants display significant phenotypic similarity to humans with Dravet syndrome, including spontaneous seizures, resistance to many available antiepileptic drugs, and early death [14,17]. The zebrafish knock-out model of neuro-hyperactivity, obtained by loss-of-function mutations in the GABA receptor subunit alpha 1 (gabra1^−/−^), offers a unique advantage for drug-screening purposes because seizures (in addition to the sporadic ones) can be triggered by exposure to light [119]. In this context, CBD and THC significantly reduced the seizure-induced total distance moved, both in chemically induced and genetic models [14,119]. Although the exact mechanisms by which cannabinoids exert their antiseizure effects are not well understood, a number of molecular targets are known to be modulated by cannabinoids. Because CBD is a positive allosteric modulator of GABA receptors, it could, for example, be capable of reducing seizure events through this mechanism. This might hold true despite the fact that THC has been associated with GABA-release inhibition [14], as, even in this case, the THC properties depend, at least in part, on the seizure-model characteristics and cannabinoid dose. Furthermore, as previously indicated, the ability of these phytocannabinoids to reduce seizures could also be mediated by the transient receptor potential vanilloid 1 (TRPV1) channel: THC, CBD, CBN, and CBDV are all transient receptor potential cation channel subfamily A member 1 agonists. Finally, NMDA receptor, glycolysis, and fatty acid amide hydrolase may be potential cannabinoid targets, participating in seizure-effect modulation [14]. Recently, a commercially available library containing 370 synthetic cannabinoids (compounds engineered to bind cannabinoid receptors with high affinity) was screened [17] in 5 dpf homozygous scn1Lab^−/−^ zebrafish larvae in order to identify molecules with the ability to reduce seizure-like behaviors. Five compounds exerting significant antiseizure activity during acute exposure were identified. It is essential to note that synthetic cannabinoids are not FDA-approved “for human or veterinary use”, and substantial evidence of serious adverse effects has been reported for some of them [17]. Further research using the above models could be of great help in discerning the true therapeutic potential of various cannabinoids for the treatment of epilepsy.

**Table 1:** An overview of cannabis-exposure effects in zebrafish.

Studies Carried Out on Embryos/Larvae						
Compound Concentration and Exposure	Strain	Age	Nonbehavioral Analysis	Behavioral Analysis	Results	References
**THC** (0.016, 0.031, 0.156, 0.469, and 0.625 mg/L) and **CBD** (0.225, 0.3, 0.525, 0.75, and 1.125 mg/L). **Acute exposure** (3–4 min before analysis).	AB/TU ^1^	5 dpf	/	VMR test: 150 min of light followed by a 5 min dark–light cycle for 30 min.	**Locomotion:** THC: decreased locomotor activity at all concentrations tested; CBD: increased locomotor activity at concentrations above 0.525 mg/L.	Achenbach et al., 2018 [31]
**THC** (2, 4, 6, 8, and 10 mg/L) and **CBD** (1, 2, 3, and 4 mg/L). **Acute exposure** (5 h during gastrulation stage).	TL ^2^	5 dpf	Survival, hatching rate, morphology, basal heart rate, and synaptic activity at neuromuscular junctions	Auditory/mechanical escape response test	**Survival:** Embryos exposed to 8–10 mg/L THC and 3–4 mg/L CBD had reduced survival rates. **Hatching rate:** Reduced with both THC and CBD, at all concentrations tested. **Morphology and basal heart rate:** Dose-dependent reductions in both body length and heart rate. **Synaptic activity:** Reduced with 6 mg/L THC and 3 mg/L CBD. **Escape response:** No reduction in touch response but decreases in sound response with 6 mg/L of THC and 3 mg/L of CBD.	Ahmed et al., 2018 [10]
**THC** (0.3–3.4 mg/L), **CP 55,940** (2.25–18 mg/L), and **WIN 55,212-2** (0.3–1.8 mg/L). **Acute exposure** (1, 4, and 12 min before analysis) and **chronic exposure** (96 h: from 24 to 120 hpf).	/	5 dpf	LD_50_ determination and morphology	VMR test: 4 min of light followed by 4 min of dark.	**LD_50_:** A total of 3.37 mg/L for THC, 1.8 mg/L for WIN 55,212-2, and 16.92 mg/L for CP 55,940. **Morphology:** THC caused malformations at all concentrations tested, while CP 55,940 and WIN 55,212-2 did not significantly increase the frequency of malformations. **Locomotion:** In acute exposure conditions, a biphasic response (stimulation at low concentrations and suppression at high concentrations) was observed; in chronic exposure, only 1.2 mg/L THC had a significant effect (increased distance traveled).	Akhtar et al., 2013 [15]
**THC** (6 mg/L). **Acute exposure** (5 h during gastrulation stage).	TL ^2^	2 and 5 dpf	Morphology of Mauthner cells and immunohistochemical analysis of the trunk muscles	Mechanical escape response test at 2 dpf and VMR test at 5 dpf (60 min).	**Morphology:** THC exposure reduced axonal diameter of Mauthner cells. **Escape response:** No reduction in C-bend response rate, but C-bend angle was increased in THC-treated embryos. **Immunohistochemistry:** White and red muscle fibers appeared thinner and slightly disorganized in THC-treated embryos. **Locomotion:** THC impaired locomotor performance.	Amin et al., 2020 [75]
**Dihydrophenanthrene derivative** (1–5 μM). **Acute exposure** (3–4 min before analysis).	AB/TU ^1^	5 dpf	/	VMR test: 150 min of light followed by 5-min dark–light cycles (for 30 min).	**Locomotion:** Locomotor activity was increased at concentrations from 2.5 to 5 μM during the first 50 min, but normally increased larval locomotor activity was reduced during the dark phases.	Banskota et al., 2021 [12]
**THC** (0.3125, 0.625, 1.25, 2.5, 5 mg/) and **CBD** (0.075, 0.15, 0.3, 0.6, 1.2 mg/L). **Chronic exposure** (94 h: from 2 to 96 hpf).	Tg(fli1:egfp)	4 dpf	Toxicity and morphology	Touch response and VMR test: 10 min light–dark cycles (for 30 min).	**Morphology:** THC and CBD displayed concentration-dependent morphological toxicities. **Locomotion:** Larvae exposed to 0.3 mg/L THC, or 0.07 mg/L CBD, exhibited a significantly increased duration of movement during dark phases compared with control. By contrast, 1.25 mg/L THC and 0.1–0.3 mg/L CBD significantly reduced duration of movement compared with control.	Carty et al., 2018 [86]
**THC** (0.024, 0.12, and 0.6 mg/L) and **CBD** (0.006, 0.03, and 0.15 mg/L). **Chronic exposure** (90 h: from 6 to 96 hpf).	Tg(fli1:egfp)	4 dpf	Survival and fertility rate	VMR test: 10 min dark–light cycles (for 30 min).	**Survival and fertility:** Not affected by treatments. **Locomotion:** Hypoactivity observed in larvae exposed to the lowest concentration of THC, and only during the dark phases.	Carty et al., 2019 [32]
**CBN** (0.25, 0.75, 1.0, 1.125, 1.2, 1.25, and 2 mg/L). **Chronic exposure** (96 h: from 24 to 120 hpf).	AB	5–7 dpf	Survival, morphology, LD_50_, and basal heart rate	VMR test: 10 min dark–light cycles (for 30 min). Mechanical escape response test: 2 min in dark conditions.	**LD_50_:** 1.12 mg/L. **Morphology:** Concentrations higher than 0.75 mg/L led to malformations. **Basal heart rate:** At concentrations higher than 0.75 mg/l, heart rate decreased significantly, exhibiting characteristic bradycardia. **Locomotion:** Distance was significantly reduced as CBN concentration increased in both dark and light conditions; velocity increased with increasing CBN concentration under dark conditions and decreased under light conditions. **Escape response:** No differences.	Chousidis et al., 2020 [30]
Acute exposure to acetic acid solution (0.1–0.5%) (nociception stimulus), followed by exposure to **THC** (0.15625 mg/L) or **CBD** (0.15 mg/L). **Acute exposure** (2 h before analysis).	AB/TU ^1^	5 dpf	/	VMR test: 2.5 h exposure to light followed by 5 min dark–light cycles (for 30 min).	**Locomotion:** THC-exposed larvae showed reduced activity compared with both acetic acid-exposed and control-group larvae, while CBD elevated the activity level of the larvae compared with acetic acid-exposed group. There was no significant reduction in the light–dark transition response in any of the test groups.	Ellis et al., 2018 [25]
**20 synthetic cannabinoids** (1, 10, and 100 μM). **Acute exposure** (20 min before analysis).	*scn1lab^−/−^*	5 dpf	Electrophysiology	VMR test: 10 min	**Electrophysiology:** Five synthetic cannabinoids decreased the frequency of spontaneous epileptiform events. **Locomotion:** Five synthetic cannabinoids decreased seizure-like swims in a concentration-dependent manner.	Griffin et al., 2020 [17]
**CBD** (0.5, 1, 5, and 10 mg/L) and **WIN55,212–2** (0.5, 1, 5, and 10 mg/L). **Acute exposure** (30 min).	/	4–6 dpf	/	VMR test: 15 min dark–light cycle for 180 min.	**Locomotion:** CBD reduced the movement velocity and total distance moved. Moreover, CBD at 10 mg/L attenuated the responses of larvae exposed to darkness. No differences were detected between the control and CBD-treated groups after 24 h in fresh water. Fish treated with WIN55,212–2 at 0.5 and 1 mg/L showed virtually no activity, even in darkness, whereas a concentration of 10 mg/L induced mortality. A 24 h period in fresh water had the effect of reversing most of the drug-induced immobilization, even in the WIN55,212-2-treated groups. Finally, treatment with CBD attenuated WIN55,212-2-induced abnormal immobilization, whereas equivalent doses of CBD and WIN55,212–2 produced a mixed response.	Hasumi et al., 2020 [111]
**THCV** (0.286 and 0.859 mg/L) and **THCV−OH** (0.859 mg/L). **Chronic exposure** (5 days: from 3 to 8 dpf).	/	8 dpf	Number of ossified vertebral centers	/	**Morphology:** THCV reduced the number of ossified vertebral centers, whereas THCV−OH increased it.	Janssens et al., 2018 [37]
**THC** (2, 3, 4, and 6 μg/mL) and **CBD** (1.5, 2, and 3 mg/L). **Acute exposure** (9.5 h: from 0.5 to 10 hpf).	CaMPARI transgenic/*Casper*	4–5 dpf	Neural activity	VMR test: 60 min	**Neural activity:** Reduced in embryos exposed to 2–3 mg/L of CBD and 4–6 mg/L of THC. **Locomotion**: Reduced in embryos exposed to 3 mg/L CBD and 6 mg/L THC.	Kanyo et al., 2021 [3]
**Whole-plant** cannabis extract. **Chronic exposure** (96 h of exposition starting at 24 hpf).	AB	5 dpf	Gene expression	VMR test: 150 min of light followed by 5 min dark–light cycles (for 30 min).	**Locomotion:** During both the first 150 min of light and the remaining 30 min of light–dark cycles, larvae treated with cannabis at the highest dose (200 µL) showed increased locomotor activity. **Gene expression:** Both zebrafish cannabinoid receptors (*cnr1* and *cnr2*) were overexpressed at the highest dose (200 µL).	Licitra et al., 2021 [91]
**WIN55,212-2** (0.014–3.412 mg/L) and **CP55,940** (0.188–3.013 mg/L), and specific *cnr2* agonists **HU-910** and **JWH-133**. **Acute exposure** (1 h before analysis).	AB/TU ^1^ and *cnr1^−/−^*	5 dpf	/	VMR test: 4 min of light, 4 min of dark, and 30 min of light.	**Locomotion:** WIN55,212-2 and CP55,940 produced a dose-dependent reduction in locomotor activity in both the light and dark phases. HU-910 and JWH-133 have no effect on locomotion. In the *cnr1^−/−^* larvae, no inhibitory effect of WIN55,212-2 or CP55,940 on the average swimming velocity was found. The *cnr1* antagonist AM251 did not affect locomotor activity, but blocked the effect of WIN55,212-2, which suggests that endocannabinoids are not active in regulating locomotor activity in zebrafish larvae at 5 dpf.	Luchtenburg et al., 2019 [87]
**Whole-plant** cannabis extracts. **Acute exposure** (2 h).	AB/TU ^1^	5 dpf	/	VMR test: 90 min of light followed by 5 min dark–light cycles (for 30 min).	**Locomotion:** During the first 30 min of light, exposure to high THC extracts led to reduced activity at 0.25 mg/L and higher activity at 1 and 2 mg/L. Instead, exposure to high CBD extracts led to hyperactivity at 0.5 and 1 mg/L. During the final 30 min of the light cycle, high THC extracts significantly decreased activity at all concentrations tested, while high CBD extracts led to a reduction in activity only at 2 mg/L. During light–dark transitions, the locomotor response was abolished at 2 mg/L (in the dark phase).	Nixon et al., 2021 [92]
**THC** (0.156–2.1875 mg/L), **CBD** (0.3–2.1 mg/L), and **THC–CBD** combination. **Acute exposure** (1 h before analysis).	AB/TU + PTZ and *GABRA1* knock-out	5 dpf	/	VMR test: 30 min	**Locomotion:** THC, CBD, and their combination reduced PTZ-induced neuro-hyperactivity and alleviated *GABRA1^−/−^* seizures.	Samarut et al., 2019 [119]
**THC** (1, 2, 5, and 10 mg/L). **Acute exposure** (19.5 h: from 4.5 to 24 hpf).	/	1–9 dpf	Survival and morphology	Tail twitches	**Survival:** After 24 h of exposure, no effects of THC on survival were recorded, but after between 2 and 9 days of exposure, survival was greatly reduced. **Morphology:** At levels above 2 mg/L THC, larvae showed curved trunks and/or bulbous-tipped tails. **Coiling:** At 26–28 hpf, the number of twitches following exposure to 5 or 10 mg/L THC was significantly reduced.	Thomas, 1975 [104]
**CBD** (0.075–0.3 mg/L), **THC** (0.3125–1.25 mg/L), **CBDV** (0.072, 0.172, 0.286, and 1.146 mg/L), **CBN** (0.078, 0.186, 0.310, and 1.242 mg/L), or **LN** (0.107, 0.256, 0.427, and 1.707 mg/L). **Acute exposure** (24 h: from 120 to 144 hpf).	*scn1lab^−/−^* and WT (*scn1lab^+/+^* or *scn1lab^+/−^* + PTZ)	6 dpf	Morphology	VMR test: 15 min	**Morphology:** CBN and CBDV led to a high incidence of deformities. **Locomotion:** THC (1.25 mg/L) significantly reduced total distance traveled. In wild-type specimens, PTZ-induced hyperlocomotion was significantly reduced following exposure to CBD or THC, but no changes were observed following CBDV, CBN, or LN exposure. In the *scn1lab^−/−^* mutants, the total distance traveled was significantly reduced following exposure to CBD (0.15 mg/L), THC (0.3125 mg/L), CBN, and LN.	Thornton et al., 2020 [14]
**O-1602** and **abnormal** **CBD** (up to 3.14 mg/L). **Chronic exposure** (3 days: from 2 to 5 dpf).	AB/TU ^1^	5 dpf	Toxicity and antitumor effects	/	**Toxicity:** Concentrations greater than 2.5 μM led to higher levels of toxicity to the larvae. **Antitumor:** Both atypical cannabinoids significantly reduced the presence of injected cancer cells in the zebrafish larvae, by approximately 50%.	Tomko et al., 2019 [24]
**CBD** (5, 20, 70, 150, and 300 μg/L). **Chronic exposure** (4 days: from 0 to 4 dpf).	/	3–4 dpf	Toxicity and morphological analysis	Motor activity calculated as number of active events for 3 min.	**Toxicity and morphology:** CBD did not show significant differences in the morphological parameters at any dose, but at the highest concentration, CBD significantly delayed the hatching time of embryos. **Locomotion:** Above 20 μg/L, CBD increases the motor activity at 24 hpf, but not at 48 hpf.	Valim Brigante et al., 2018 [52]
**Studies carried out in adult fish**						
**THC** (0.024, 0.12, and 0.6 mg/L) and **CBD** (0.006, 0.03, and 0.15 mg/L). **Chronic exposure** (90 h: from 6 to 96 hpf).	Tg(fli1:egfp)	12–18 months	Reproductive parameters	Open field test: 6 min	**Reproduction:** Reduced fecundity in adults exposed to CBD (0.15 mg/L) and THC (0.024 and 0.12 mg/L). **Locomotion:** No significant effects.	Carty et al., 2019 [32]
**WIN55,212-2** (0.5, 5, or 50 mg/L). **Acute aqueous exposure** (10 min) and **dietary exposure** (1 μg/day/fish for 1 week).	/	/	/	Light–dark cross-maze test: 5 min	**Locomotion:** Altered behavioral anxiolytic responses and reduced locomotor activity at all tested doses. A 1-week dietary exposure promoted zebrafish exploration.	Connors et al., 2014. [125]
**THC** (0.0125, 0.3125, and 0.625 mg/L). **Acute exposure** (2 min prior to starting analysis).	EK	9–12 months	/	Locomotion: 20 min	**Locomotion:** THC (0.3125 mg/L) reduced velocity and induced repetitive swimming patterns.	Dahlén et al., 2021 [2]
**CBD** (40 mg/L). **Acute exposure** (30 min).	/	6 months	Gene expression	Locomotion: 3 min	**Locomotion:** CBD reduced distance traveled and velocity. **Gene expression:** CBD activated genes encoding proinflammatory cytokines (*il-1b* and *il-17a/f2*).	Jensen et al., 2018 [7]
**CBD** (0.1 0.5, 5.0, or 10 mg/kg) via **intraperitoneal injection** (1 h before analysis).	TU	4 months	/	Locomotion, anxiety, aggressive behavior (1 min), and social interaction (10 min). Memory-assessment task.	**Locomotion:** Not affected. **Anxiety:** Inverted U-shaped dose–response curve with 0.5 mg/kg reducing the anxiety. **Aggressive behavior and social interaction**: Not affected by 5 mg/kg CBD. **Memory:** CBD (5 mg/kg) caused memory impairment.	Nazario et al., 2015 [48]
**THC** (0.024, 0.12, and 0.6 mg/L). **Chronic exposure** (90 h: from 6 to 96 hpf).	Tg(fli1:egfp)	12–30 months	Survival, reproductive and growth parameters, and gene expression	Open field: 5 min	**Survival:** Increased at 0.024 mg/L THC in male fish. A significant reduction in survival of F1 THC-treated male fish by 30 months of age. **Reproduction:** THC exposition did not significantly alter sperm production, and exposure to 0.024 mg/L THC improved egg production in aged females; the resulting offspring at 96 hpf showed similar survival to both young and aged control fish. The F1 fish parentally exposed to 0.6 mg/L THC were completely unable to reproduce, unlike the aged controls. **Growth:** No difference in body length or mass was observed in male fish exposed to vehicle or THC, while exposure to the lowest concentration of THC (0.024 mg/L) resulted in significant reductions in mass in advanced aged females. **Locomotion:** With the exception of increased mobility in 0.12 mg/L-THC-exposed males, early-life treatment with THC did not affect locomotor abilities in 30-month-old male or female fish. **Gene expression:** Significant reductions in *tnf-α* and *il-1β,* and increases in *il-6*, *pparα* and *pparγ*.	Pandelides et al., 2020 [27]
**THC** (0.03125 mg/L). **Acute exposure** (1 h).	/	12 months	/	Color-discrimination learning and spatial-cognition task.	**Color-discrimination learning:** THC administration did not lead to any observable effect on color-discrimination learning. **Spatial cognition:** Impaired.	Ruhl et al., 2014 [113]

^1^ Tübingen; ^2^ Tübingen longfin.

## 4. Pointers on Behavioral Analysis

Behavioral analysis was performed in 18 of the 21 studies dealing with zebrafish larvae. The age of the larvae ranged from 1 to 7 dpf. Two tests were applied: the VMR test and the mechanical escape response; the latter was used in three of the 18 studies and was combined with an auditory stimulus in only one of them [10]. The VMR test normally involves several phases of light–dark succession, and it aims to stimulate an unconscious defensive response initiated by a drastic change in lighting [126]. In wild-type larvae without sight impediments, the locomotor activity increases at light onset, before decreasing to the baseline level after ca. 30 s. The wild type also shows increased locomotor activity at light offset, but they need more time (ca. 30 min.) to return to the baseline level of locomotion [127]. During embryogenesis, mechanical stimulus to the tail of the zebrafish embryo can be used to elicit the coiling behavior (touch response) [128]. Similarly, the escape response can be stimulated in larvae using mechanical, acoustic, electrical, or optical stimuli [129]. The escape response mimics predator-avoidance behavior, which is usually mediated by the Mauthner cells [130] located in the hindbrain [125,131].

Of the five studies carried out in zebrafish adults, four evaluated locomotion, one of these also explored social behavior and memory [48], and the other was conducted on color-discrimination learning and spatial cognition [113].

Overall, the results on the locomotion, both in larvae and adult fish, showed significant differences between studies. Cannabinoids, depending on the concentrations used, could either increase or decrease locomotor activity. As we stated in a recent systematic review on social-preference tests in zebrafish [101], the lack of a standardized approach to behavioral assessment makes it difficult to compare studies. Furthermore, in view of the heterogeneity in terms of the administered cannabinoids, doses, and exposure times of the current research, the standardization of behavioral tests could help to allow inferences to be drawn from findings in zebrafish species and provide more consistent data for translational-medicine purposes. The age of larvae used to perform the VMR test could, ideally, be set at 5 dpf: at this age, the larvae show limited (but sufficient) physiological development [132], but they are not yet independently feeding and are therefore subject to the EU directive on the protection of animals used for scientific purposes (Directive 2010/63/EU). Moreover, the exposure time could be set at 24 hpf, or 120 hpf to evaluate the effects of prolonged exposure. It should be highlighted that the daily replacement of the drug was performed in only one of the studies reviewed [37]. Although the adsorption of the medication can be considered minimal, and especially in the case of cannabinoids [3,10], we still believe that an approach that keeps the drug concentration constant over time, and that also considers the possible evaporation of egg water or medical compound, if volatile, will be the most accurate. The approach could be further standardized by introducing a standard duration of locomotor experiments and choosing the preferred drug-administration route for studies in adult zebrafish. We think the duration should be 30 min, and that drugs could be optimally administered through food. Furthermore, with regard to the method used to analyze the behavioral effects of cannabinoid treatments in adults, it may be useful to elect the novel tank test as the major read-out, considering that behavioral experiments should ideally last 10 min, after 5 min of habituation time.

## 5. Conclusions

This review showed that the zebrafish may prove a useful model for cannabinoid translational research because it displays similar behaviors to rodents following cannabinoid exposure. Moreover, it is clearly necessary to pay more attention to the full spectrum of naturally occurring cannabinoids, rather than focusing on the main ones: THC and CBD. These results indicate a need for additional cannabis-based studies to shed light on the mechanistic properties of cannabinoids, and to provide insight into the potential risks of its therapeutic application. At the same time, it is necessary to consider the long-term consequences of early-life exposure to cannabinoids.

## Data Availability

Not applicable.
